# Racial-Ethnic Differences in Fall Prevalence among Older Women: A Cross-Sectional Survey Study

**DOI:** 10.1186/s12877-017-0447-y

**Published:** 2017-03-11

**Authors:** Yifan Geng, Joan C. Lo, Leslea Brickner, Nancy P. Gordon

**Affiliations:** 1 Department of Medicine, Kaiser Permanente Oakland Medical Center, Oakland, CA USA; 2 Division of Research, Kaiser Permanente Northern California, 2000 Broadway, Oakland, CA 94612 USA

**Keywords:** Falls, Race/ethnicity, Older women, Risk factors

## Abstract

**Background:**

Falls are the leading cause of hip fracture in older women, with important public health implications. Fall risk increases with age and other clinical factors, and varies by race/ethnicity. International studies suggest that fall risk is lower in Asians, although data are limited in U.S. populations. This study examines racial/ethnic differences in fall prevalence among older U.S. women within a large integrated healthcare delivery system.

**Methods:**

This cross-sectional study used data from 6277 women ages 65–90 who responded to the 2008 or 2011 Kaiser Permanente Northern California Member Health Survey (KPNC-MHS). The KPNC-MHS is a mailed questionnaire sent to a random sample of adult members stratified by age, gender, and geographic location, representing a population estimate of >200,000 women age ≥65 years. Age, race/ethnicity, self-reported health status, presence of diabetes, arthritis or prior stroke, mobility limitations and number of falls in the past year were obtained from the KPNC-MHS. The independent association of race/ethnicity and recent falls was examined, adjusting for known risk factors.

**Results:**

The weighted sample was 76.7% non-Hispanic white, 6.2% Hispanic, 6.8% black and 10.3% Asian. Over 20% reported having fallen during the past year (28.5% non-Hispanic white, 27.8% Hispanic, 23.4% black and 20.1% Asian). Older age was associated with greater fall risk, as was having diabetes (OR 1.24, CI 1.03–1.48), prior stroke (OR 1.51, CI 1.09–2.07), arthritis (OR 1.61, CI 1.39–1.85) and mobility limitations (OR 2.82, CI 2.34–3.39), adjusted for age. Compared to whites, Asian (OR 0.64, CI 0.50–0.81) and black (OR 0.73, CI 0.55–0.95) women were much less likely to have ≥1 fall in the past year, adjusting for age, comorbidities, mobility limitation and poor health status. Asians were also less likely to have ≥2 falls (OR 0.62, CI 0.43–0.88).

**Conclusions:**

Among older women, the risk of having a recent fall was substantially lower for black and Asian women when compared to white women. This may contribute to their lower rates of hip fracture. Future studies should examine cultural and behavioral factors that contribute to these observed racial/ethnic differences in fall risk among U.S. women.

## Background

The incidence of falls in older Chinese individuals residing in Asia is substantially lower than that reported in older white populations [1]. Comparison of fall rates examined across Asia, Europe and Australia suggest that fall rates in Asians are lower compared to other racial/ethnic subgroups, despite similarities in other fall risk factors [1–3]. Limited historical data based on findings among Japanese residents in Hawaii [2, 4, 5] and recent data published from the 2011–2012 California Health Interview Survey [6] also indicate that fall rates appear to be lower in U.S. Asians compared to whites and/or non-Asians. Understanding these racial/ethnic differences is crucial as the proportion of Asians and other minority subgroups are expected to increase substantially and become a larger percentage of U.S. seniors in the coming years [7]. While less than one in ten falls results in fracture [8], clinical fractures and especially hip fractures remain a major public health concern for older individuals, the latter with an annual incidence of 0.8% per year and mortality of over 20% in women age 65 and older [9, 10]. The incidence of hip fracture similarly varies by race, with rates of hip fracture notably lower among Asian American [11–13] and black women [11, 12] compared to non-Hispanic white women. However, unlike black women who have higher bone mineral density than women of white race [14], Asian women typically have lower bone mineral density [15, 16], suggesting that other factors beyond bone mineral density contribute to ethnic differences in hip fracture, including risk of falls. Prevention of falls is an important component of hip fracture risk reduction [17]. This study examines the prevalence of falls and differences by age and race/ethnicity among older women identified from a large Northern California integrated healthcare delivery system.

## Methods

### Study aim and design

The aim of this cross-sectional study is to describe the racial/ethnic differences of older women in relation to fall prevalence and relevant social, behavioral and reported medical conditions of these women, using data collected from a large population mailed survey.

### Data source and participants

This cross-sectional study was conducted using data from 6,277 non-Hispanic white (*n* = 4,705), black (*n* = 463), Hispanic (*n* = 425), and Asian (*n* = 684) women health plan members ages 65–90 who responded to the 2008 or 2011 Kaiser Permanente Northern California (KPNC) Member Health Survey (MHS) and provided information about the number of times they had fallen in the prior 12 months [18]. The MHS is conducted every 3 years using a confidential self-administered (print or online) questionnaire sent to an age-, gender-, and geographically stratified random sample of KPNC adult members. The survey collects self-reported information about a variety of member characteristics, including sociodemographic data, selected health conditions, overall health status, health-related behaviors/lifestyle risks, and for seniors, falls and functional status. More detailed information about the Member Health Survey can be found elsewhere [18]. In both 2008 and 2011, the survey response rate for women ages 65–90 was 69%. Previous analyses have shown that the KPNC adult population is highly comparable to the population of insured adults in Northern California with regard to demographic and health-related characteristics, with the exception of a smaller percentage of adults at very low levels of income and education [19]. The MHS and descriptive studies based on MHS data were approved by KPNC’s Institutional Review Board.

### Study variables

The primary outcome variable for this study, falls in the past 12 months, was ascertained from the question “In the past 12 months, how many times have you fallen to the ground or fallen on stairs?” The comorbidities (diabetes, stroke, arthritis) were derived from responses to checklists of conditions the person ever had, was treated for, or had taken medications for, and overall health status was assessed using the question “In general, would you say your health is: Excellent, very good, good, fair, or poor.” Individuals were considered to have a significant mobility limitation if they indicated needing the help of a cane, walker, wheelchair, or another person to get around in the house or outside, or needed to stay in the house most of the time versus having no limitations or no limitations that required help from another person or special aid.

### Statistical analyses

All analyses were conducted used pooled (2008 + 2011) MHS respondent data weighted with a post-stratification weighting factor to better reflect the actual age (by 5-year intervals), gender and geographic composition of the adult health plan membership in 2011. Prior to pooling and weighting, we deleted the 2008 data for 61 women found to have participated in both survey years. While percentages reported in the text and tables are based on weighted data, column Ns in the tables are the unweighted subgroup denominators. Fall rates and health characteristics were calculated for non-Hispanic white, black, Hispanic, and Asian women aged 65–90 using the Proc Surveymeans procedure in PC-SAS version 9.3 for data collected using a complex survey design (SAS Institute, Inc., Cary, NC) [20]. SAS Proc Surveylogistic was used to test for significant racial/ethnic differences in health characteristics and in having fallen in the past 12 months, after adjusting for age group (65–74, 75–79, 80–85 and 85–90 years old), and multivariable logistic regression models were used to examine the association of race/ethnicity and having fallen in the past year, after adjusting for age group, comorbidities, poor health status, and significant mobility limitation. Unadjusted and adjusted odds ratios (ORs) are shown with 95% confidence intervals (CI).

## Results

A total of 6,277 women aged 65–90 years old (mean age 74.4 years) who self-identified as one of the four racial/ethnic groups completed the survey and provided information about falls in 2008 or 2011. The cohort, after weighting, was 76.7% non-Hispanic white, 6.2% Hispanic, 6.8% black, and 10.3% Asian. On average, the non-Hispanic white women were older and more likely to report very good or excellent health and less likely to have diabetes when compared to black, Hispanic and Asian women (Table 1). Black women had a higher incidence of stroke compared to whites, and both blacks and Hispanics had a higher proportion needing mobility assistance. Rates of self-reported arthritis were similar by race. The prevalence of having at least one fall in the past year was 28.5% for non-Hispanic white women, 27.8% for Hispanic and 23.4% for black women, and 20.1% for Asian women. Adjusting for differences in age, Asian women remained significantly less likely than women of non-Hispanic white race to report having at least one fall in the past year (OR 0.65, CI 0.52–0.83), and were also less likely to have had 2 or more falls in the prior year (OR 0.64, CI 0.45–0.90).

**Table 1:** Clinical characteristics and history of recent falls among women ages 65–90 years old, by race/ethnicity

	Non-Hispanic White	Black	Hispanic	Asian
Characteristic	*N* = 4749	*N* = 465	*N* = 428	*N* = 696
	Wtd. % (95% CI)	Wtd. % (95% CI)	Wtd. % (95% CI)	Wtd. % (95% CI)
Age, mean (95% CI)	74.6 (74.4–74.9)	73.7 (72.9–74.5)^a^	73.5 (72.6–74.3)^a^	73.5 (72.9–74.2)^b^
Age group (years)				
65–74	53.9% (52.2–55.6)	60.1% (54.8–65.3)^a^	61.1% (55.5–66.7)^a^	60.1% (55.7–64.4)^b^
75–79	18.5% (17.6–19.4)	18.1% (15.2–21.0)	19.2% (16.0–22.4)	19.5% (17.0–22.1)
80–84	15.5% (14.1 = 16.9)	14.3% (9.8–18.7)	10.3% (6.2–14.3)	11.5% (8.0–14.9)
85–90	12.1% (10.7–13.5)	7.6% (4.0–11.1)	9.4% (4.9–14.0)	8.9% (5.7–12.1)
Overall health status				
Very good/excellent	41.5% (39.8–43.2)	23.3% (18.8–27.8)^c^	29.1% (24.0–34.2)^d^	32.4% (28.2–36.5)^d^
Good	39.7% (38.0–41.3)	44.8% (39.4–50.2)	42.1% (36.5–47.7)	45.4% (40.8–49.9)
Fair	16.1% (14.9–17.4)	28.9% (23.8–33.9)	25.2% (19.8–30.6)	18.9% (15.4–22.4)
Poor	2.6% (2.1–3.2)	3.0% (1.3–5.6)	3.5% (1.1–5.6)	3.3% (1.5–5.1)
Diabetes mellitus	12.8% (11.7–13.9)	30.1% (25.0–35.2)^c^	23.1% (18.3–27.8)^c^	20.7% (17.0–24.5)^c^
Stroke	4.4% (3.7–5.2)	7.4% (4.5–10.3)^d^	5.7% (2.6–8.8)	2.9% (1.6–4.2)
Arthritis	31.4% (29.8–33.0)	31.8% (26.8–36.8)	28.3% (23.1–33.6)	29.2% (25.1–33.3)
Mobility				
No limitations	75.4% (73.9–76.9)	73.8% (68.9–78.7)	73.0% (67.4–78.5)	74.5% (70.3–78.6)
Some limitation	8.3% (7.3–9.2)	6.4% (3.9–9.0)	7.7% (4.6–10.8)	10.9% (7.9–14.9)
Needs assistive device (cane, etc.) or help from someone to get around	16.4% (1510–17.7)	19.8% (15.2–24.3)^d^	19.3% (14.2–24.4)^e^	14.6% (11.2–17.9)
Number of falls in past year				
1 or more falls	28.5% (26.9–30.1)	23.4% (18.8–27.9)	27.8% (22.5–33.1)	20.1% (16.5–23.7)^c^
2 or more falls	12.7% (11.6–13.8)	11.6% (8.2–15.0)	15.3% (10.8–19.7)	8.2% (5.8–10.6)^d^

*Wtd*. % weighted percent based on data weighted to the study population, 95% *CI* confidence interval around the weighted percentage, unadjusted for age

All statistics are based on self-reported survey data, with denominators restricted to women with falls data

^a^Significantly different from non-Hispanic white using logistic regression at *p* < 0.05

^b^Significantly different from non-Hispanic white using logistic regression at *p* < 0.01

^c^Significantly different from non-Hispanic white after controlling for age group using logistic regression at *p* < 0.001

^d^Significantly different from non-Hispanic white after controlling for age group using logistic regression at *p* ≤ 0.01

^e^Significantly different from non-Hispanic white after controlling for age group using logistic regression at *p* < 0.05

**Fig. 1:**
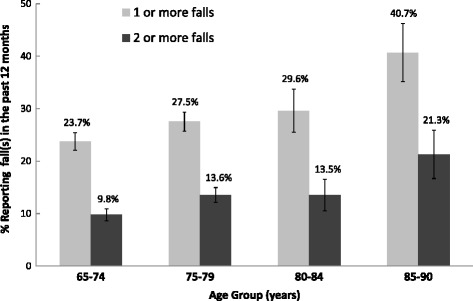
The prevalence of one or more self-reported falls in the past 12 months in older women, stratified by age. Percentages with 95% confidence intervals are shown

History of one or more falls in the past 12 months increased significantly by age (Fig. 1), with the odds of having a fall in the past year 2-fold higher for women age 85–90 compared to women age 65–74 years old (OR 1.22, CI 1.07–1.39 for ages 75–79 years; OR 1.35, CI 1.09–1.68 for ages 80–85 years, and OR 2.20, CI 1.72–2.82 for ages 85–90). A similar increased risk by age was seen for two or more falls in the past year. In addition, statistically significant associations were found between fall prevalence and comorbidities of interest and mobility status. Presence of diabetes (OR 1.24, CI 1.03–1.48), prior stroke (OR 1.51, CI 1.09–2.07), arthritis (OR 1.61, CI 1.39–1.85), self-reported poor health (OR 2.56, CI 1.71–3.84) and mobility limitation (OR 2.82, CI 2.34–3.39) were associated with an increased risk of having a fall in the past year, adjusted for age. In multivariable logistic regression analyses, adjusting for differences in age, comorbidity, self-reported poor health status, and mobility limitations, Asian (adjusted OR 0.64, CI 0.50–0.81) and black (adjusted OR 0.73, CI 0.55–0.95) women were significantly less likely to have had at least one fall in the past year compared to non-Hispanic white women (Table 2). Asian women were similarly less likely to have had two or more falls in the past year (adjusted OR 0.62, CI 0.43–0.88), whereas the adjusted risk for black women no longer significantly differed from non-Hispanic whites.

**Table 2:** Adjusted fall prevalence among different racial/ethnic groups

	Unadjusted	Age-adjusted	Age-adjusted + comorbidities^a^	Age-adjusted + comorbidities^a^ + poor health	Age-adjusted + comorbidities^a^ + poor health + mobility limitation
Outcome: ≥ 1 fall in past year	OR (95% CI)	OR (95% CI)	OR (95% CI)	OR (95% CI)	OR (95% CI)
Non-Hispanic white	Reference	Reference	Reference	Reference	Reference
Black	0.77^b^	0.80	0.75^b^	0.76^b^	0.73^b^
	(0.59–1.00)	(0.61–1.04)	(0.57–0.99)	(0.58–0.99)	(0.55–0.95)
Hispanic	0.97	1.00	0.98	0.98	0.94
	(0.74–1.27)	(0.76–1.32)	(0.74–1.30)	(0.74–1.30)	(0.71–1.24)
Asian	0.63^c^	0.65^c^	0.65^c^	0.64^d^	0.64^c^
	(0.50–0.80)	(0.52–0.83)	(0.51–0.82)	(0.50–0.82)	(0.50–0.81)
Outcome: ≥ 2 falls in past year	OR (95% CI)	OR (95% CI)	OR (95% CI)	OR (95% CI)	OR (95% CI)
Non-Hispanic white	Reference	Reference	Reference	Reference	Reference
Black	0.90	0.95	0.86	0.87	0.85
	(0.64–1.27)	(0.67–1.34)	(0.60–1.24)	(0.60–1.26)	(0.59–1.23)
Hispanic	1.24	1.30	1.26	1.26	1.21
	(0.87–1.77)	(0.90–1.87)	(0.87–1.83)	(0.87–1.83)	(0.83–1.76)
Asian	0.61^d^	0.63^d^	0.63^d^	0.62^d^	0.62^d^
	(0.44–0.86)	(0.45–0.90)	(0.45–0.90)	(0.43–0.88)	(0.43–0.88)

*OR* adjusted odds ratio, 95% *CI* confidence interval around the adjusted odds ratio

^**a**^Comorbidities include diabetes, arthritis and history of stroke

^b^Significantly different from non-Hispanic white after controlling for age group using logistic regression at *p* < 0.05

^c^Significantly different from non-Hispanic white after controlling for age group using logistic regression at *p* < 0.001

^d^Significantly different from non-Hispanic white after controlling for age group using logistic regression at *p* ≤ 0.01

We next examined subgroups of Asian women, given potential physical, health-related and cultural differences among Asians by ethnic origin. Most (92%) of the Asians identified in our study could be grouped into two subgroups, East Asians of Chinese, Korean, and Japanese ethnic origin (*n* = 321) and Filipinas (*n* = 308). The two subgroups did not differ significantly in mean age (74.2 vs. 73.2, respectively, *p* = 0.15), but East Asians were less likely than Filipinas to have a history of diabetes (14.8% vs. 26.7%, *p* = 0.003), arthritis (22.7% vs. 35.9%, *p* = 0.003), poor health (1.5% vs. 5.5%, *p* < .004), and mobility limitations (9.2% vs. 21.2%, *p* < 0.001); the groups did not differ in history of stroke (3.1% vs. 2.7%, *p* = 0.74). East Asian women tended to be more likely than Filipina women to have had a fall in the past 12 months (23.7% vs. 17.2%, *p* = .098). After adjusting for age, comorbidities, health status, and mobility limitations, this difference became statistically significant (adjusted *OR* = 2.04, CI 1.21–3.45). Furthermore, when we specifically examined Chinese women, given potential cultural influences in physical activity (e.g. practice of Tai Chi or other balance and flexibility exercises), we found that Chinese women remained more likely than Filipinas to have had a fall in the past 12 months (adjusted *OR* = 1.93, CI 1.07–3.49).

## Discussion

Among older women residing in northern California, Asian and black women were significantly less likely than non-Hispanic white women to have fallen in the past 12 months, and Asians, but not blacks, were also significantly less likely to have fallen two or more times in the past 12 months. These results support recent data from the 2011–2012 California Health Interview Survey, which surveyed 13,897 older adults (including 1193 Asians) and found that 12.2% of the population (but only 7.6% of Asians) were estimated to have fallen at least twice in the past year [6]. In this same study, non-Asians had a 1.7-fold higher adjusted odds of multiple falls in the past year compared to non-Hispanic Asians [6]. These findings also support prior observations of lower hip fracture rates among Asian and black women compared to white women residing in the U.S. [11–13], and international data demonstrating lower incidence of falls in Asian compared to white populations [2, 21]. However, East Asian women were significantly more likely than Filipinas to have fallen in the past 12 months, despite a lower prevalence of fall-related comorbidities, suggesting that differences in fall risk may exist among Asian subgroups. We also found that having at least one fall was significantly associated with older age, diabetes, and stroke, and mobility limitations, consistent with the known relationship of these comorbidities with an increased risk of falls [22–27].

Falls occur in up to 30% of older community-dwelling U.S. seniors each year [8]. The risk factors for falls include older age, female gender, previous history of falls, vision problems, gait difficulties, conditions limiting physical function, relevant comorbidities and psychotropic or other medication exposures [8, 22, 28]. Identifying patients at risk is important, given mounting evidence that intervention can reduce fall rates [8] and potentially address the high economic burden and poorer quality of life associated with hip fractures. Multiple studies suggest that fall rates vary among different racial/ethnic subgroups [1, 4, 29–31], including lower rates among Asian compared to Caucasian women residing in the same region [5]. Potential reasons for these differences vary and may include heritable, cultural, health-related or behavioral factors that could influence both the tendency and risk of falling [15, 29]. The mechanics of falling also vary by race and impact the risk of subsequent fracture. For example, white women are more likely to fall laterally and sustain a fracture, whereas black women are more likely to fall forward and less likely to sustain a fracture [29]. Cultural factors such as concern about falling and Tai chi exercise are also possible explanations of why Chinese women in Asia have lower rates of falls [2], although the extent to which these cultural influences apply to Chinese women residing in the U.S. is less clear. In our study, Asian women paradoxically reported more mobility limitations, but they also reported less use of assistive devices and had lower fall prevalence. This may suggest that Asian women are self-limiting certain activities based on their own perceived limitations and this may be reducing their fall risk; however, we cannot exclude the possibility of under-reporting of falls among Asians [1, 6]. Rates of hip fracture in California are highest for white women and lower for black and Asian women [11, 30], with mortality post-hip fracture also lower for Asian women [9]. National data similarly demonstrate that age-adjusted hip fracture rates among Asian women are 2-fold lower than those among white women [12]. The results of our study suggest that lower fall risk among U.S. Asians in relation to other ethnic populations may help explain their lower hip fracture rate.

Our study does have some limitations to consider. First, there are inherent limitations of survey methodology with variable response rates and selection bias due to the fact that only patients able to communicate in written English were included, requiring some degree of acculturation. Length of residence within the U.S. and country of origin were also not specifically examined in this study. Second, there may be recall bias in the reporting of falls and other self-reported health characteristics, and linkage to health records for ascertainment of relevant comorbidities, medication use and other risk factors was not examined. Third, these data represent cross-sectional associations and cannot be used to determine causation. Finally, differences in prevalent falls were observed among East Asians (and Chinese) women compared to Filipinas, emphasizing the importance of further examination of specific ethnic subgroups across larger populations, given the heterogeneity of the Asian American population. The strengths of our study include examination of a large population cohort with over 6000 women age 65 and older, ability to identify Asian ethnic groups, and a survey response rate of nearly 70%. This weighted population sample is also highly representative of the Northern California insured population of up to 225,520 women age 65–90 years and provides contemporary data on prevalent falls and associated risk factors in a multi-ethnic population. Within the overall U.S. population, approximately 5.6% are Asian, increasing to 14.9% within California [32].

## Conclusions

As the U.S. population continues to age and more people become at risk for falls and subsequent hip fracture, a greater understanding of disparities in fall risk is vital for future prevention efforts. Our study quantifies racial ethnic differences in fall prevalence among older U.S. women residing in northern California and demonstrates that Asian Americans and African Americans overall appear less prone to falling compared to white non-Hispanics, consistent with international reports. Further studies should investigate the potential mechanisms, including social and cultural factors, underlying these ethnic differences. In the interim, these findings may assist clinicians in understanding culturally specific approaches to evaluating a patient’s fall risk while also informing broader prevention efforts.

## Abbreviations

BMD: Bone mineral density
CI: Confidence interval
KPNC: Kaiser Permanente Northern California
KPNC MHS: Kaiser Permanente Northern California Member Health Survey
MHS: Member Health Survey
OR: Odds ratio
PC-SAS: Statistical Analysis System for personal computers
U.S: United States
